# Corticospinal excitability modulation in resting digit muscles during cyclical movement of the digits of the ipsilateral limb

**DOI:** 10.3389/fnhum.2015.00607

**Published:** 2015-11-04

**Authors:** Tetsuro Muraoka, Masanori Sakamoto, Nobuaki Mizuguchi, Kento Nakagawa, Kazuyuki Kanosue

**Affiliations:** ^1^College of Economics, Nihon UniversityTokyo, Japan; ^2^Faculty of Education, Department of Physical Education, Kumamoto UniversityKumamoto, Japan; ^3^Faculty of Sport Sciences, Waseda UniversitySaitama, Japan; ^4^Graduate School of Sport Sciences, Waseda UniversitySaitama, Japan

**Keywords:** TMS, interlimb coordination, fingers, toes, grasping, corticospinal excitability

## Abstract

We investigated how corticospinal excitability of the resting digit muscles was modulated by the digit movement in the ipsilateral limb. Subjects performed cyclical extension-flexion movements of either the right toes or fingers. To determine whether corticospinal excitability of the resting digit muscles was modulated on the basis of movement direction or action coupling between ipsilateral digits, the right forearm was maintained in either the pronated or supinated position. During the movement, the motor evoked potential (MEP) elicited by transcranial magnetic stimulation (TMS) was measured from either the resting right finger extensor and flexor, or toe extensor and flexor. For both finger and toe muscles, independent of forearm position, MEP amplitude of the flexor was greater during ipsilateral digit flexion as compared to extension, and MEP amplitude of the extensor was greater during ipsilateral digit extension as compared to flexion. An exception was that MEP amplitude of the toe flexor with the supinated forearm did not differ between during finger extension and flexion. These findings suggest that digit movement modulates corticospinal excitability of the digits of the ipsilateral limb such that the same action is preferred. Our results provide evidence for a better understanding of neural interactions between ipsilateral limbs, and may thus contribute to neurorehabilitation after a stroke or incomplete spinal cord injury.

## Introduction

Movement of one limb enhances not only corticospinal excitability of muscles employed in the movement but also the excitability of resting muscles of other limbs (Carson et al., 1999; Baldissera et al., 2002; Borroni et al., 2004; Byblow et al., 2007; Mcintyre-Robinson and Byblow, 2013). For example, when a wrist is cyclically extended and flexed, corticospinal excitability of the contralateral resting wrist extensors and flexors increased during the extension and flexion, respectively (Carson et al., 1999). That is, wrist movement of one limb enhances the corticospinal excitability of homologs muscles of the other limb. As to the relationship between hand and foot muscles, though there are no homologs muscles, similar modulation of the corticospinal excitability can be obtained from hand muscles during ipsilateral foot movement. When cyclically moving the foot upward (dorsiflexion) and downward (plantarflexion), corticospinal excitability of the ipsilateral resting wrist extensors and flexors increased during dorsiflexion and plantarflexion, respectively, with the forearm in a pronated position on an armrest (Borroni et al., 2004). When the forearm is in a supinated position, wherein activation of wrist extensors produces downward movement of the hand, the corticospinal excitability of wrist extensors increases during plantarflexion, but not dorsiflexion (Borroni et al., 2004). Thus, the excitability of wrist muscles is modulated so that it favors the same directional movement of hand and foot, not simultaneous activation of a specific coupling of wrist and ankle muscles. The corticospinal excitability modulation of one limb induced by other limb movement could be utilized as an add-on therapy to physical rehabilitation exercises after stroke to modulate damaged brain region (e.g., bilateral movement training; Stewart et al., 2006; Summers et al., 2007; Morris et al., 2008). In particular, recovery of digit function is quite beneficial for stroke patients and has been shown to improve their quality of life (Dettmers et al., 2005). However, little is known how digit movement of the upper limb affects the corticospinal excitability of digits of the lower limb, and* vice versa*.

One of the main functions of the digits involves grasping, although human toes are rarely used this way in daily life. On the other hand, one of the main functions of the wrist and ankle is to locate the hand and foot in external space in the proper position to attain a desired goal. This difference in function would be expected to be related to different control mechanisms for the involved joints. Grasping requires the translation of visual information regarding the physical properties of an object into motor commands, and an object-centered reference frame independent of external space is used in this process (Patchay et al., 2006). In addition, the space surrounding the hands is encoded in a hand-centered reference frame in the premotor and posterior parietal cortices (Brozzoli et al., 2012). On the other hand, the transportation movements such as reaching with more proximal joints need translation of visual information in external space into motor commands, and thus an external reference frame is used (Patchay et al., 2006). Movement direction in an external reference frame predominantly determines the stability of coordinated movements of the wrist and ankle (Baldissera et al., 1982; Carson et al., 1995; Salesse et al., 2005; Muraoka et al., 2013). However, this is not the case for coordinated movements of bilateral fingers (Riek et al., 1992) or that of digits of the ipsilateral limbs (i.e., fingers and toes; Muraoka et al., 2015). Since there is a difference between the reference frame utilized for digit and proximal joint movement, the possibility also exists that the modulation pattern for corticospinal excitability of resting wrist muscles induced by ipsilateral ankle movement may not be applicable to digits of the ipsilateral limbs.

The present study therefore aimed to investigate how corticospinal excitability of the resting digit muscles was modulated by digit movement of the ipsilateral limb. In particular, we evaluated whether the excitability modulation was dependent upon movement direction in external space or the action of digits. We accomplished this by using pronated and supinated forearm positions. If the excitability is modulated on the basis of the direction of movement in external space, digit movement should induce a different excitability of digit muscles of the ipsilateral limb, depending upon whether the forearm is in the pronated or supinated position. Alternatively, if the excitability is modulated on the basis of action of the digits, digit movement should induce a similar excitability of digit muscles of the ipsilateral limb irrespective of forearm position. Corticospinal excitability was assessed by measuring motor evoked potentials (MEPs) elicited by transcranial magnetic stimulation (TMS) over the primary motor area (M1). We hypothesized that the pattern of excitability modulation of the resting digit muscles during digit movement of the ipsilateral limb was independent of movement direction in external space. Rather, it would be dependent upon “grasping action” (i.e., specific muscle coupling of the finger and toe) in order to enhance the same action of the digits of the ipsilateral limbs.

## Materials and Methods

### Subjects

Seventeen normal male subjects (21–33 years) were used for the measurement of MEPs from the finger muscles. Eight subjects from this experiment were used for the measurement of MEPs from the toe muscles. Nine new subjects were added so that the latter experiment also had a total of seventeen subjects (20–34 years). No subject had history of neurological disease. All subjects were fully informed about the purpose of the study and its procedures. Written informed consent was obtained from all subjects. This study was approved by the Human Research Ethics Committee of the Faculty of Sport Sciences, Waseda University.

### Experimental Setup

**Figure 1:**
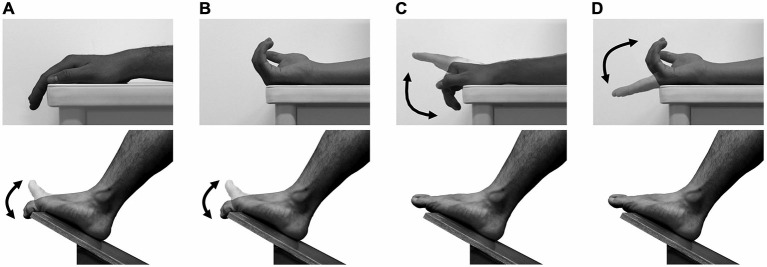
**Fingers and toes movements.** The TMS was delivered over the primary motor area of finger muscles during toe extension/flexion with the forearm either pronated **(A)** or supinated **(B)** and over the primary motor area of toe muscles during finger extension/flexion with the forearm either pronated **(C)** or supinated **(D)**. The two directional arrows indicate the movement of fingers or toes.

Subjects sat in a chair with the right forearm supported in a horizontal position on an armrest. The right foot was put on an inclined board with the toes in the air (Figure 1). The angular displacements of the middle phalanx of the right index finger relative to the dorsum of the right hand and those of the distal phalanx of the right second toe relative to the dorsum of the right foot were measured at 4 kHz using electrical goniometers (SG150, Biometrics, UK). The goniometers were taped on the dorsum of the hand and of the middle phalanx of the index finger or the dorsum of the foot and of the distal phalanx of the second toe. The joint angular signals were stored on a computer via an AD converter (PowerLab16/30, ADInstruments, Australia), and then were low-pass filtered with a cutoff frequency of 10 Hz.

### Electromyography (EMG)

Electromyography (EMG) signals were recorded at 4 kHz from a finger extensor muscle (extensor digitorum communis, EDC), a finger flexor muscle (flexor digitorum superficialis, FDS), a toe extensor muscle (extensor digitorum brevis, EDB), and a toe flexor muscle (flexor digitorum brevis, FDB). After careful abrasion of the skin, bipolar surface Ag-AgCl electrodes (20 mm interelectrode distance) were placed on the center of each muscle belly of the right limbs. The EMG signals were bandpass filtered (5–1500 Hz), digitized, and stored on the computer via the AD converter.

### Transcranial Magnetic Stimulation (TMS)

For cortical activation of the finger muscles, M1 of the left hemisphere was stimulated using a magnetic stimulator (Nihon Kohden, model SMN-1200, JPN) connected to a round coil (outer diameter, 140 mm) centered over the vertex, with a maximum intensity of 0.67 T. For the toe muscles, M1 of left hemisphere was stimulated using a magnetic stimulator (Magstim Company Ltd., model Magstim-200, UK) connected to a double cone coil (outer diameter of each half coil, 110 mm) placed over the leg motor area (2–3 cm posterior to the vertex and slightly left of the midline, long axis of the intersection of the half coils pointing forwards, parallel to the mid-line), with a maximum intensity of 1.3 T. We stimulated M1, moving the coil in rostro-caudal and medio-lateral directions, and selected two positions on the scalp where we could elicit low threshold short latency MEP from both EDC and FDS or EDB and FDB. The positions of the coil were marked on the scalp with a pen and these same coil positions were utilized throughout the experiment. The coils were held manually. The resting threshold was defined as the lowest stimulus intensity that produced at least five of ten consecutive MEPs with amplitude of 50 μV in the less excitable muscle while both EDC and FDS or EDB and FDB were at rest (Rossini et al., 1994). The test stimulus intensity was set at 110% of the resting threshold.

### Protocols and Analysis

The subjects were instructed to perform cyclical extension-flexion movements either of the right toes or of the right fingers at 0.75 Hz prescribed by an auditory metronome beat of 1.5 Hz while keeping right finger or toe muscles relaxed, respectively. MEPs were obtained either from the resting finger extensor (EDC) and flexor (FDS) during toe movements or from the resting toe extensor (EDB) and flexor (FDB) during finger movements. The finger and toe movements represent a “grasping action” of the four fingers or five toes using proximal and distal interphalangeal joints, and metacarpophalangeal or metatarsophalangeal joints (Figure 1). In order to eliminate a potential influence of visual stimuli (i.e., observing moving digits) on modulation of the MEP, the subjects were instructed to keep their eyes closed. The right forearm was maintained in either a pronated or in a supinated position. After each trial was verbally initiated, the metronome beat started and the subjects were instructed to initiate the cyclical movements. The subjects were instructed to continue the movements until TMS was delivered when the toes or fingers reached about two thirds of the range of motion (ROM) of either the extension or flexion phase within the third to fifth cycle. At this point the EMG activities of the agonists were relatively high through the cycle for all four muscles investigated. The stimulation timing was randomized. Before the experiment, the subjects were asked to perform finger or toe movement to the beat of the metronome. Then, an appropriate interval between metronome beat and TMS for each subject was identified and used in the experiment. In each experiment, for either MEP measurement of finger or toe muscles, each subject underwent 80 trials, 20 MEPs for each condition (total four conditions: 2 movements (extension, flexion) × 2 forearm positions (prone, supine)). The order of the forearm position was counterbalanced across subjects. The inter-trial interval was set to more than 10 s, thus the interval between two successive stimulations was at least 15 s. The subjects were instructed to keep the finger or toe muscles completely relaxed throughout the trials for the MEP measurement of toe or finger muscles, respectively. To aid in the attainment of complete relaxation, the right forearm and palm, and the right foot were firmly fixed to the armrest and footrest by tape. Trials in which background EMG activities of the target muscles for the MEP measurement were greater than 25 μV within the last 50 ms prior to stimulation were excluded from further analysis. It was assumed that in this case, the subjects did not maintain the target muscles in a relaxed state.

Peak-to-peak amplitude of MEP was measured and averaged for each condition for each muscle. Subsequently, data on the MEP amplitude was submitted to a two-way analysis of variance (ANOVA) with repeated measures using movement (extension vs. flexion), and forearm position (prone vs. supine). When there was an interaction between movement × forearm position, a paired *t*-test with the Bonferroni correction was used to test for differences between the MEP amplitude during extension and flexion for each forearm position. Background EMG activity was calculated as the root mean square (RMS) values of EMG activity recorded during the 50 ms prior to each TMS. The influence of forearm position on digit position where MEPs were obtained was tested by a paired *t*-test with the Bonferroni correction. Values are presented as means ± SD. Statistical significance was set at a level of *p* < 0.05.

## Results

### MEP Amplitude of Finger Muscles

**Figure 2:**
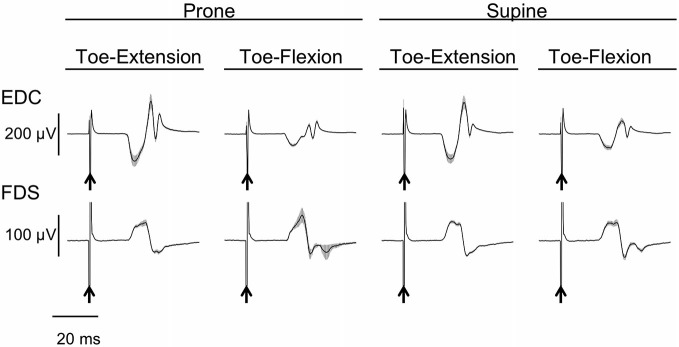
**Typical example of motor evoked potential (MEP) of finger extensor and flexor.** Mean and SD of 20 trials for each task from one subject. The arrows indicate the timing of the TMS. The finger extensor is the extensor digitorum communis (EDC) and the flexor is the digitorum superficialis (FDS).

**Figure 3:**
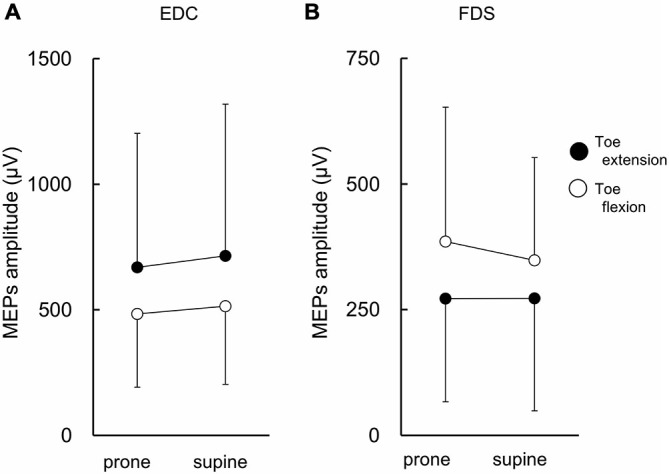
**MEP amplitude of finger extensor and flexor.** The finger extensor is the extensor digitorum communis (EDC) and the flexor is the digitorum superficialis (FDS). There were significant main effects of toe movements on MEP amplitude for both EDC **(A)** and FDS **(B)**. Values are mean +/− SD. *N* = 17.

The MEPs in EDC and FDS taken from a single subject are shown in Figure 2. The MEPs amplitudes of EDC and FDS tended to be greater when they were elicited during toe extension and flexion, respectively. The average MEPs amplitudes in 17 subjects are shown in Figure 3. Statistical analysis revealed that there was a significant main effect of movement (*F*_(1,16)_ = 4.94, *p* = 0.041) on MEP amplitude of the finger extensor, whereas neither a main effect of forearm position nor an interaction between movement × forearm position was significant (*F*_(1,16)_ = 0.30, *p* = 0.593 and *F*_(1,16)_ = 0.62, *p* = 0.443, respectively). This indicates that MEP amplitude of EDC was greater during toe extension than during toe flexion irrespective of forearm position. Similarly, there was a significant main effect of movement (*F*_(1,16)_ = 5.09, *p* = 0.038) on MEP amplitude of the finger flexor. Neither a main effect of forearm position nor an interaction between movement × forearm position was significant (*F*_(1,16)_ = 2.57, *p* = 0.128 and *F*_(1,16)_ = 0.67, *p* = 0.424, respectively). This indicates that MEP amplitude of FDS was greater during toe flexion than during toe extension irrespective of forearm position.

### MEP Amplitude of Toe Muscles

**Figure 4:**
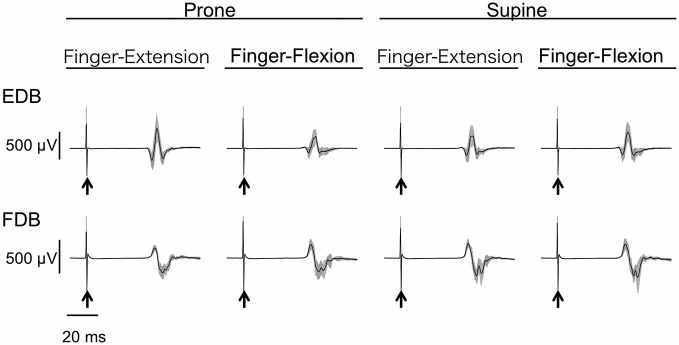
**Typical example of MEP of toe extensor and flexor.** Mean and SD of 20 trials for each task from one subject. The arrows indicate the timing of the TMS. The toe extensor is the extensor digitorum brevis (EDB) and the flexor is the flexor digitorum brevis (FDB).

**Figure 5:**
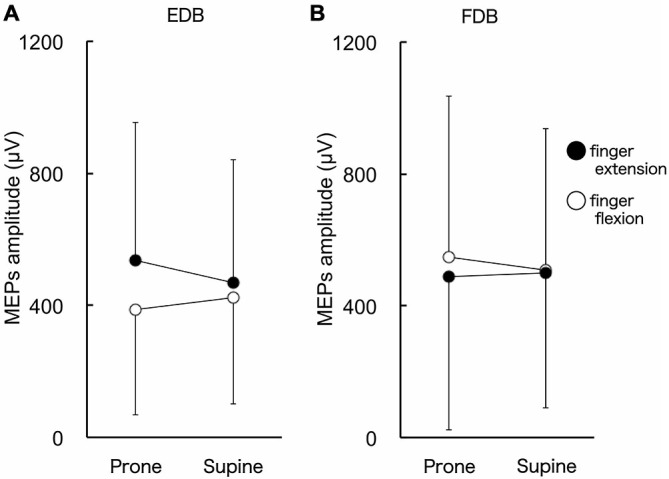
**MEP amplitude of toe extensor and flexor.** The toe extensor is the extensor digitorum brevis (EDB) and the flexor is the digitorum brevis (FDB). There were significant main effects of finger movements on MEP amplitude of EDB **(A)**. MEP amplitude of FDB was greater during finger flexion than extension when the forearm was in the pronated position **(B)**. Values are mean +/− SD. *N* = 17.

The MEPs in EDB and FDB taken from a single subject are shown in Figure 4. The MEPs amplitudes of EDB and FDB tended to be greater when they were elicited during finger extension and flexion with the forearm in the pronated position, respectively. The average MEPs amplitudes in 17 subjects are shown in Figure 5. Statistical analysis revealed that there was a significant main effect of movement (*F*_(1,16)_ = 8.25, *p* = 0.011) on MEP amplitude of the toe extensor, whereas neither a main effect of forearm position nor an interaction between movement × forearm position was significant (*F*_(1,16)_ = 0.12, *p* = 0.737 and *F*_(1,16)_ = 2.44, *p* = 0.137, respectively). This indicates that MEP amplitude of EDB was greater during finger extension than during finger flexion. There was a significant main effect of movement on MEP amplitude of the toe flexor (*F*_(1,16)_ = 6.43, *p* = 0.022) and a significant interaction between movement × forearm position (*F*_(1,16)_ = 5.22, *p* = 0.036). Further analysis for movement in each forearm position showed that MEP amplitude of FDB was significantly greater during finger flexion than during finger extension with the forearm in the pronated position (*t*_(16)_ = 2.81, corrected *p* = 0.026), but not in the supinated position (*t*_(16)_ = 0.61, uncorrected *p* = 0.546). No significant main effect of forearm position on MEP amplitude of the toe flexor was observed (*F*_(1,16)_ = 0.18, *p* = 0.675).

### TMS Conditions

Stimulation intensity for finger and toe muscles were 69 ± 13% and 64 ± 11% of the maximal output of the magnetic stimulator with the round coil and the double cone coil. Of the total 2720 trials, 162 trials (7%) were excluded from the formal analysis because of the presence of background EMG activity. The background EMG activities in the muscles from which MEPs were obtained were slightly different across muscles (EDC: 10 μV, FDS: 8 μV, EDB: 4 μV, FDB: 4 μV), but were constant over the four conditions for each muscle (the difference among conditions was less than 1 μV on average). MEPs were obtained at 67 ± 14% ROM (0% and 100% ROM corresponded to peak toe/finger flexion and extension, respectively) during toe extension and at 33 ± 13% ROM during toe flexion. For finger extension and finger flexion the figures were 67 ± 12% ROM and 29 ± 10% ROM. No significant influence of forearm position on digit position where MEPs were obtained (*t*_(16)_ = 1.52, 1.34, 0.41, and 0.25; uncorrected *p* = 0.148, 0200, 0.685, and 0.810 for finger flexion, finger extension, toe flexion, and toe extension, respectively) was observed. Since the EMG activities of the agonists around two thirds of ROM were relatively high through the cycle for all four muscles investigated, the background EMG activities that were obtained from moving digit were greater in EDC during finger extension (185 ± 29 μV) than flexion (29 ± 19 μV), in FDS during finger flexion (46 ± 32 μV) than extension (19 ± 10 μV), in EDB during toe extension (67 ± 56 μV) than flexion (22 ± 22 μV), and in FDB during toe flexion (42 ± 42 μV) than extension (17 ± 15 μV).

## Discussion

The fundamental goal of this study was to investigate the principles that describe how limb movements influence corticospinal excitability modulation of the resting muscles of the ipsilateral limb. Previous studies have shown that ankle movements modulate corticospinal excitability of the wrist muscles such that same directional movements of the hand and foot are preferred (Baldissera et al., 2002; Borroni et al., 2004; Byblow et al., 2007; Mcintyre-Robinson and Byblow, 2013). However, it is unknown as to whether this kind of direction related neural interactions exist between digits in the ipsilateral limbs. The present study measured the MEP of resting digit muscles during digit movement of the ipsilateral limb. The results indicated that corticospinal excitability of the resting digit muscles was modulated so that the same action of the digits of the ipsilateral limbs (i.e., simultaneous flexion or extension of fingers and toes) was preferred, although toe flexor muscle did not show such modulation when the forearm was in the supinated position.

### Corticospinal Excitability Modulation of Resting Digit Muscles During Digit Movement of the Ipsilateral Limb

The MEP amplitude of the resting finger flexor was greater during ipsilateral toe flexion as compared to toe extension irrespective of forearm position (Figure 3). Similarly, the MEP amplitude of the resting finger extensor and toe extensor was greater during toe and finger extension, respectively (Figures 3, 5). The MEP amplitude of toe flexor was greater during finger flexion when the forearm was in the pronated position, although finger movement did not produce an excitability modulation of the toe flexor when the forearm was in the supinated position (Figure 5). That is, in seven of eight conditions (4 muscles × 2 forearm positions), the MEP amplitude of the resting digits was greater when the muscle with a similar function in the ipsilateral limb (i.e., flexor or extensor) was active as an agonist as compared to when it was less active as an antagonist. This is definitely different from the modulation pattern of wrist muscles during ankle movement in which movement direction is the dominant factor for the modulation (Borroni et al., 2004; Marconi et al., 2007). Therefore, it is suggested that if corticospinal excitability modulation of resting digit muscles during digit movement of the ipsilateral limb occurs, it occurs in such a way that the same action of the digits of the ipsilateral limbs is preferred. The difference in neural mechanisms between previous studies and the present study are discussed in more detail below (see “Principle of excitability modulation of resting muscles during joint movements of ipsilateral limb” section).

The action dependent excitability modulation is a unique feature of the digits. Is it possible to infer that finger movements with the supinated forearm did not produce the excitability modulation of the toe flexors because toe flexors might have partially lost the characteristics of digits? As a result of evolution, human fingers have become capable of performing fine movements. This dexterity must, perforce, have been accompanied by the development of highly sophisticated direct corticospinal connections (Nakajima et al., 2000). At the same time, human toes apparently lost dexterity as typified by the non-opposable hallux. Indeed, Brouwer and Ashby (1992) showed that direct corticospinal connections to toe muscles were less than those to tibialis anterior muscle and suggested that it reflected a loss of dexterity of the toes in humans. However, it should be noted here that they also showed that direct corticospinal connections were relatively greater in FDB compared to EDB. The present results showed the modulation of the corticospinal excitability of EDB during finger movement is independent of movement direction, but dependent on a “grasping” action. It follows that the density of direct corticospinal connections and loss of dexterity cannot explain why only FDB did not show modulation of corticospinal excitability when the forearm was in the supinated position. Further studies are needed to investigate why finger movements with the supinated forearm did not induce corticospinal excitability modulation in toe flexor muscles.

Several studies have revealed that mental practice with motor imagery could be beneficial as an add-on intervention to standard physical therapies in order to activate partially damaged motor networks (Page et al., 2001; Stevens and Stoykov, 2003; Butler and Page, 2006). Nudo et al. (1996) showed that in monkeys, rehabilitative physical training of the hand after a focal ischemic infarct in the hand area of M1 prevented a further loss of hand area in the adjacent undamaged area and reorganized the undamaged area so that fine hand function was regained. Since regaining digit function would greatly improve the quality of life (Anderson, 2004; Dettmers et al., 2005), the present results suggest a possible add-on intervention to neurorehabilitation after a stroke or incomplete spinal cord injury, like mental practice with motor imagery. For example, rehabilitation of finger grasping for patients with paralyzed fingers might be more effective if toe grasping with any forearm posture were simultaneously executed.

### Principle of Excitability Modulation of Resting Muscles During Joint Movements of Ipsilateral Limb

It has been shown that corticospinal excitability of the resting wrist muscles was modulated by ipsilateral ankle movement such that the same directional movement of hand and foot was preferred (Borroni et al., 2004; Marconi et al., 2007) independent of the particular coupling of joint actions. However, the present study showed that corticospinal excitability of finger and toe muscles was largely modulated during repetitive flexion and extension of the ipsilateral toes and fingers so that the same action, and not the same direction, was preferred. Does the modulation in wrist muscles and digit muscles function according to different principles? It should be noted here that one of the main functions of the wrist and ankle is to position the hand and foot at a discrete location in external space, while that of the digits involves “grasping action”. Thus the results of the previous studies and the present one could be unified into a single principle: corticospinal excitability is modulated to enhance a common salient function (i.e., moving limbs in the same direction or performing the same action) of the ipsilateral joints (muscles). Moreover, this principle might act as one of the neural substrates for the coordination of ipsilateral limbs. The coordination stability of ipsilateral wrist and ankle is predominantly determined by movement direction (Baldissera et al., 1982; Carson et al., 1995; Salesse et al., 2005; Muraoka et al., 2013), and indeed Mcintyre-Robinson and Byblow (2013) showed a significant correlation between stability of wrist-ankle coordinated movement in the opposite direction and the extent of modulation of the corticospinal excitability that supported same directional movement. On the other hand, a previous study of our group showed that action coupling as well as movement direction predominantly determine the coordination stability of the ipsilateral finger and toe (Muraoka et al., 2015), and the present study showed modulation of corticospinal excitability that supported the same action of digits of the ipsilateral limbs. To investigate the causal relationship between coordination stability and the extent of the excitability modulation that supports the same action of digits of the ipsilateral limbs is a challenge for the future.

### Neural Networks for the Excitability Modulation

The H-reflex modulation of resting wrist muscle during ankle movement that supported same directional movement of the wrist and ankle disappeared when it was tested in the cortical silent period induced by TMS (i.e., 40–60 ms after conditioning; Baldissera et al., 2002). Thus it was suggested that excitability modulation of the wrist muscle during ankle movement occurred at the cortical level, not at the spinal level. In addition, the excitability modulation was tightly linked to ankle muscle EMG, not to ankle kinematics (Cerri et al., 2003), and occurred during imagined ankle movements as well (Marconi et al., 2007). Therefore parallel excitability modulations may occur in both ankle and wrist M1 during ankle movement with resting wrist muscle. Since there are no direct anatomical connections between ankle and wrist M1 (Huntley and Jones, 1991), higher-order motor areas must be involved in such parallel excitability modulations. In support of this, Byblow et al. (2007) investigated how a conditioning TMS over the dorsal premotor cortex (PMd) and rhythmic ankle movement affected excitability modulation in wrist muscle M1. They suggested that a neural network between PMd and M1 would likely be responsible for the parallel excitability modulations (Byblow et al., 2007). There is a possibility that a PMd-M1 network is also responsible for the excitability modulation between finger and toe muscles if the excitability modulations both between hand and foot and between finger and toe are based on the principle we stated above (see “Principle of excitability modulation of resting muscles during joint movements of ipsilateral limb” section). In support of this, PMd uses a joint (muscle) based reference frame as well as an external reference frame (Wu and Hatsopoulos, 2007), and PMd is one of the regions recruited during “grasping” (Filimon, 2010). In future work it would be interesting to investigate whether the neural interactions demonstrated in this study originate in PMd.

## Conclusion

The present study found that the movement of digits modulates corticospinal excitability of digits of the ipsilateral limb in such a way that same action is preferred. This extends our current understanding of the neural interaction between ipsilateral limbs, and may contribute to the efficacy of neurorehabilitation after stroke or incomplete spinal cord injury.

## Author Contiributions

TM designed and conducted the experiments, and wrote the manuscript. MS, NM and KN conducted the experiments. KK supervised the project and wrote the manuscript.

## Funding

This work was supported by JSPS KAKENHI Grant Number 21700660.

## Conflict of Interest Statement

The authors declare that the research was conducted in the absence of any commercial or financial relationships that could be construed as a potential conflict of interest.
